# Potential Biochemical Mechanisms of Lung Injury in Diabetes

**DOI:** 10.14336/AD.2016.0627

**Published:** 2017-02-01

**Authors:** Hong Zheng, Jinzi Wu, Zhen Jin, Liang-Jun Yan

**Affiliations:** ^1^Department of Pharmaceutical Sciences, UNT System College of Pharmacy, University of North Texas Health Science Center, Fort Worth, TX 76107, USA; ^2^Department of Basic Theory of Traditional Chinese Medicine, College of Basic Medicine, Shandong University of Traditional Chinese Medicine, Jinan, Shandong Province, 250355, China

**Keywords:** diabetic hyperglycemia, diabetic lung injury, diabetes mellitus, mitochondria, oxidative stress

## Abstract

Accumulating evidence has shown that the lung is one of the target organs for microangiopathy in patients with either type 1 or type 2 diabetes mellitus (DM). Diabetes is associated with physiological and structural abnormalities in the diabetic lung concurrent with attenuated lung function. Despite intensive investigations in recent years, the pathogenic mechanisms of diabetic lung injury remain largely elusive. In this review, we summarize currently postulated mechanisms of diabetic lung injury. We mainly focus on the pathogenesis of diabetic lung injury that implicates key pathways, including oxidative stress, non-enzymatic protein glycosylation, polyol pathway, NF-κB pathway, and protein kinase c pathway. We also highlight that while numerous studies have mainly focused on tissue or cell damage in the lung, studies focusing on mitochondrial dysfunction in the diabetic lung have remained sketchy. Hence, further understanding of mitochondrial mechanisms of diabetic lung injury should provide invaluable insights into future therapeutic approaches for diabetic lung injury.

Diabetes mellitus (DM), characterized by persistent blood hyperglycemia, is a leading cause of morbidity and mortality in the world. A recent report released by the World Health Organization reveals that there were 1.5 million (2.7%) deaths caused by diabetes in 2012, up from 1.0 million (2.0%) in 2000. The major cause of death in diabetic patients is glucotoxicity-induced complications. There are now increasing evidence showing that lung is also one of the target organs for diabetic microangiopathy in patients with either type 1 or type 2 DM [1-3]. Because the lung microvascular system has huge reserve function, diabetic lung damage is quite subclinical and often ignored by patients and physicians. With continued increase in the occurrence of diabetes in an aging population, more and more pulmonary dysfunction is likely to be attributed to diabetic pulmonary complications. Pulmonary disease associated with diabetes includes a predisposition to infections and chronic obstructive pulmonary disease such as pneumonia, asthma, pulmonary fibrosis, and pulmonary tuberculosis as well as impaired breathing during sleeps [4-11]. Furthermore, it has been reported that incidence death due to pulmonary diseases among Japanese diabetic patients has been found to be greater than 50% [12]. When compared with healthy subjects, patients with type 1 or type 2 DM are at increased risk for respiratory tract infections and the risk further increases with repeated occurrence of common infections [13, 14]. Relative risks of developing pulmonary tuberculosis of all types and bacteriologically confirmed cases were 3.47 times and 5.15 times higher, respectively, in diabetic patients than in matched healthy controls [15-18].

Therefore, elucidating the pathogenesis of the diabetic lung injury has become an important research topic. Several concepts of pathogenesis such as oxidative stress, non-enzymatic glycation of proteins, and the polyol pathway have been identified to be involved in the etiology of diabetic lung injury. In this paper, we attempt to provide an overview of the potential and major biochemical mechanisms of morphological changes and pulmonary dysfunction implicated in diabetic lung injury. A deeper understanding of the underlying mechanisms should provide invaluable insights into novel approaches for attenuating diabetic lung injury in the future. It should be noted that this review is by no means to exhaust all the possible mechanisms of diabetic lung injury documented in the literature.

## Morphological changes in diabetic lung injury

Numerous studies indicate physiological and structural abnormalities in diabetic lungs. A histological investigation in rabbit lung shows that diabetic rabbits exhibit morphological abnormalities within 3 weeks of diabetes induction [19]. It has been reported that diabetic hyperglycemia damages the respiratory system due to the pulmonary interstitial injury caused by microangiopathy, which in the meantime could also contribute to autonomic neuropathy [20]. It has also been reported that there is a widely increase in the volume proportion of alveolar wall and alveoli per unit volume, in the relative amounts of collagen, elastin, and basal laminae, and in the surface-to-volume ratio of the lungs of the diabetic rats [21]. The basal membranes were thickened, along with an intense inflammatory reaction in diabetic lungs [22]. The structures of lung tissue and lamellar bodies showed collapse in DM group [23], neutrophil infiltration or aggregation and alveolar wall thickening in lung tissue were significantly higher in the DM group than in the control group [24]. In diabetic lung models, histological examination with Sirius red and eosin and hemotoxylin staining showed fibrosis along with massive inflammatory cell infiltration [25]. The number of tiny villus and the quantity of osmiophilic multilamellar body decreased markedly, while hyperplasia was found in collagen fiber [26]. The mechanisms underlying morphological changes in diabetic lung injury might be the following: (1) Activation of NADPH oxidase that mediates oxidative and nitrosative damage [25]. (2) Activation of the polyol pathway that is one of the most popular candidate mechanisms to explain the cellular toxicity of diabetic hyperglycemia. When glucose concentration in the cell becomes too high, aldose reductase is activated to reduce glucose to sorbitol [27]. Sorbitol can induce cellular osmotic pressure, leading to cell death [28]. On the other hand, as the polyol pathway also consumes NADPH and produces NADH, cellular redox imbalance can occur and trigger oxidative stress, leading to changes in cell membrane integrity and function. (3) Generation of advanced glycation end-products (AGEs) that can impair protein structure and function.

## Pulmonary dysfunction in diabetic lung injury

Decreased lung function is associated with diabetes, both cross-sectionally and longitudinally [29-33]. It has been found that pulmonary function abnormalities appeared within 3 years of diabetes diagnosis in 51.2% of children with type 1 diabetes [34] and there is also a statistically significant increase in total airway resistance in children with type 1 diabetes [35]. Airflow restriction is a predictor of demise in type 2 diabetes after adjusting for other recognized risk factors [36]. One study in the Strong Heart Study involving 2,396 adults shows a significantly decreased pulmonary function in American Indians with DM; moreover, lung functional impairment was found to already appear in this ethnic group before the development of overt DM [37].

### Lung ventilatory dysfunction

Ventilation is an important reflection of lung function. Diabetic patients exhibit a high risk of pulmonary disorders that are often associated with restrictive impairment of lung function. Many cross-sectional studies have consistently shown that when compared with healthy subjects, patients with diabetes have significant decreases in many parameters including lower vital capacity, medium expiratory flow, expiratory residual volume, the total lung capacity (TLC), forced vital capacity (FVC), and forced expiratory volume in one second (FEV1) [38-41][26, 27]. It has also been reported that the restrictive, but not the obstructive ventilatory dysfunction, is associated with development of prediabetes and precedes the development of type 2 diabetes [42]. Moreover, it has been found that the early stage of diabetes modulates the bronchial reactivity to both acetylcholine and isoproterenol by disrupting the nitric oxide (NO), K_ATP_ channels, and cyclooxygenase pathways in guinea pigs [43]. There have been several hypotheses proposed on the mechanism of ventilatory dysfunction in the diabetic lung: (1) Respiratory muscle strength decreased, which was significantly related to attenuated lung volumes and ill metabolic control in type 2 diabetes [44-46]. (2) The restrictive type of pulmonary impairment is likely due to non-enzymatic glycosylation of pulmonary proteins and subsequent accumulation of collagen in the lungs and chest wall [30]. Moreover, glycosylation of elastin fibers can also cause improper cross-linking and emphysema-like diminution in alveolar surface area, resulting in stiffening of the lungs, reduced elastic recoil, impaired vascular diffusion, and inflammatory changes in lungs [47, 48]. (3) Nearly one-third of subjects with diabetes showed abnormal ventilatory response to hypoxia, hypercapnia, or exercise, a symptom of autonomic neuropathy [20]. Moreover, neuroadrenergic bronchopulmonary denervation may also occur in diabetic patients with autonomic neuropathy [49]. Indeed, it has been reported that neuroadrenergic denervation of the lung is associated with the decline of respiratory functional indexes in diabetic patients [50], leading to lung diastolic stress disorder [49]. (4) Non-adrenergic non-cholinergic neurotransmitter release is decreased due to diabetic autonomic neuropathy [51], which could deregulate the pulmonary vascular tone and pulmonary ventilation.

### Pulmonary diffusing capacities

Transfer of oxygen and carbon dioxide over the alveolocapillary membrane is the main function of the lung. Lung diffusing capacity for carbon monoxide (DLCO) is a measure of gas conductance across alveolar tissue membrane into capillary erythrocytes and subsequent chemical binding to hemoglobin. DLCO is influenced by alveolar-capillary membrane conductance and pulmonary capillary blood volume, both of which are reduced in adults with type 1 diabetes [52]. Evidence shows that children with type 1 diabetes also had impaired pulmonary functions including DLCO, airway resistance (Raw), alveolar volume (AV), and DLCO/AV ratio [53]. It has been demonstrated that there is a significant decrease in the ratio between DLCO and alveolar ventilation in pulmonary gas exchange in the diabetic group when compared with that in healthy controls [54]. DLCO is predominantly due to a low membrane diffusing capacity (DMCO) [55] while in type 2 diabetes both DMCO and capillary blood volume are reduced [38]. Interestingly, at peak exercise, type 1 diabetic subjects demonstrated a decreased DLCO when corrected for cardiac output (DLCO/Q) [56]. Additionally, a decrease in DMCO when corrected for cardiac output (DMCO/Q) was also present and the decrease in diffusing capacity was associated with a reduction in oxygen saturation. It is suggested that the limitation in gas transfer becomes functionally important as the transit time of red blood cells through the lung is shortened [56].

**Figure 1.**
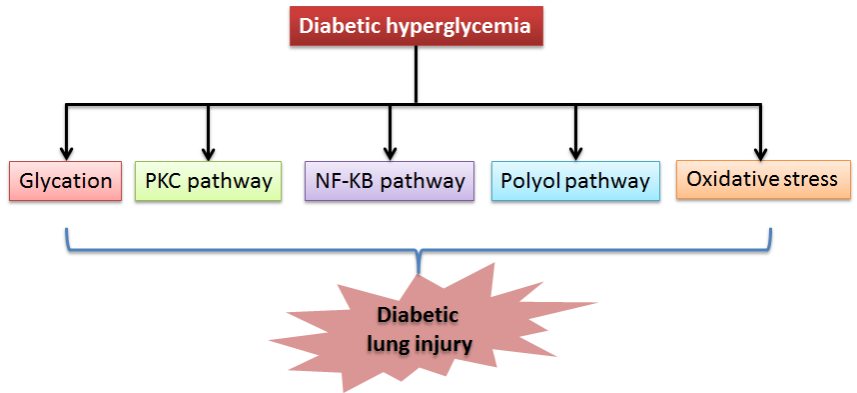
**Hyperglycemia-upregulated pathways that are potentially involved in diabetic lung injury**. These include protein glycation, PKC pathway, NF-KB pathway, polyol pathway, and oxidative stress. It should be noted that these pathways may be inter-related. For example, the polyol pathway can also contribute to oxidative stress.

## Etiopathogenesis of diabetic lung injury

There is increasingly a large body of supporting data that may provide mechanisms of pathogenesis of diabetic lung injury. However, a detailed elucidation of each mechanism remains challenging. Long lasting hyperglycemia triggers upregulation of a variety of pathways, including oxidative stress, non-enzymatic glycation of proteins, polyol pathway and sorbitol production, NF-κB pathway, and activation of the protein kinase C pathway (Fig. 1). Here we review these mainstream mechanisms that have been implicated in diabetic lung injury.

### Oxidative stress

Previous experimental and clinical studies indicate that hyperglycemia-induced overproduction of superoxide plays a key role in the pathogenesis and development of diabetic complications in all kinds of tissue injuries [57-59]. Sustained hyperglycemia produces excessive reactive oxygen species (ROS) and reactive nitrogen species (RNS) that cannot be overwhelmed by antioxidants, resulting in damage to DNA, lipids, and proteins [60-63]. Evidence indicates that in diabetic lung, the activity of superoxide dismutase (SOD) was decreased, while the contents of nitric oxide (NO) and malondialdehyde (MDA) were significantly increased [23, 26]. As NO and superoxide can react simultaneously to produce peroxynitrite that is very damaging [64], any evidence that NO concentration is elevated in the diabetic lung would suggest that NO/peroxynitrite pathway is a mechanism by which lung is injured. The pulmonary lipid peroxidation, glutathione peroxidase activities and inducible nitric oxide synthase (iNOS) were all found to be increased in streptozotocin diabetic rats [65]. Immunohistochemical staining in the pulmonary bronchial epithelium and capillary endothelium in the DM group indicates an elevated level of iNOS expression [65]. It has also been shown that 8-OHdG increases in patients with type 2 diabetes [66], demonstrating the occurrence of DNA damage in the diabetic lungs. Clinic data illustrates that tumor necrosis factor-alpha (TNF-α), interleukin-6 (IL-6), interleukin-10 (IL-10), and MDA levels in serum were all significantly increased in diabetic patients who show diabetic lung injury [67]. Additionally, it has been reported that in type 2 diabetes patients with pneumonia, levels of toll-like receptor 2/4 in peripheral blood monocytes and serum surfactant protein A were altered [68]. All these studies demonstrate the involvement of oxidative damage in diabetic lung injury.

### Non-enzymatic protein glycosylation

Nonenzymatic glycosylation is accelerated by oxidative stress and elevated levels of aldoses [69]. AGEs are a heterogeneous group of modified proteins, lipids and nucleic acids arising from intracellular hyperglycemia via a non-enzymatic Maillard reaction [63]. This modification can result in changes in tissue/cellular properties by forming crosslinks [70] that impair the biological functions of the target proteins [71]. Tissues exposed to continuous state of high blood glucose can exhibit non-enzymatic glycosylation of proteins, whereby AGEs are eventually formed [72]. It has been suggested that formation and accumulation of AGEs are involved in the pathogenesis of diabetic vascular complications [73]. As reactive oxygen intermediates are involved in the formation of AGE structures such as carboxymethyllysine [74], the interaction of AGE modified proteins with macrophages might further induce macrophage ROS production, thereby contributing to the development of pulmonary fibrosis [75].

Therefore, hyperglycemia-accelerated formation of nonenzymatic advanced glycosylation end products in tissues has been suggested as the central pathologic features of diabetic complications [76]. Interaction of AGEs with the receptor for advanced glycation end-products (RAGE) has been shown to activate down-stream signaling pathways and evoke inflammatory responses in vascular wall cells [77, 78]. Glycation can upregulate transforming growth factor-β intermediate and lead to increased synthesis of collagen, laminin, and fibronectin in the extracellular matrix [79, 80].

### Polyol pathway

Polyol pathway is one of the major sources of ROS production in diabetes [81]. Hyperglycemia decreases NAD^+^ levels by activation of the polyol pathway and by over-activation of poly (ADP-ribose)-polymerase (PARP) [82]. The pulmonary arteries from diabetic rats showed impaired relaxant response to acetylcholine and decreased vasoconstrictor response to N-omega-nitro-L-arginine methyl ester (L-NAME) that is a widely used NOS inhibitor. It has also been suggested that diabetes induces pulmonary artery endothelial dysfunction by enhancing superoxide production [83]. In experimental diabetic lung in rabbits, it was found that glutathione peroxidase (GSH-Px) activity, glutathione reductase (GSSG-R) activity, and ascorbic acid level decreased while the concentration of lipid peroxidation products increased [84]. Therefore, it is conceivable that the lowered level of NAD^+^ in the diabetic lung may aggravate cellular and tissue damage. The levels of free 15-F_2t_-isoprostane were increased in lung tissues in diabetic rats along with a significantly increased membrane translocation of the NADPH oxidase subunit p67phox and increased expression of the membrane-bound subunit p22phox in diabetic lung [85]. Additionally, damaged mitochondria can also generate an excess of superoxide that can mediate tissue injury in diabetes. Studies conducted in diabetic rats have revealed significant generation of mitochondrial superoxide at the site of NADH/ubiquinone oxidoreductase (complex I) [86, 87].

**Figure 2.**
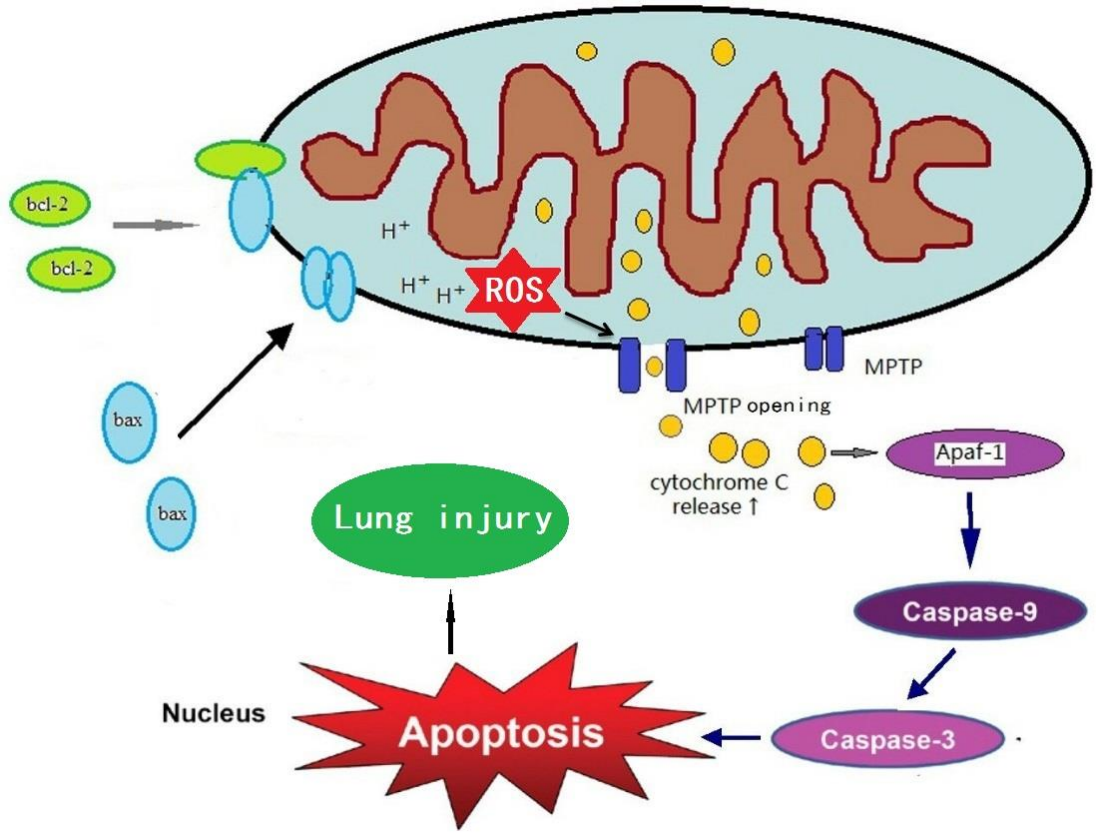
**Possible role of mitochondrial dysfunction in diabetic lung injury. Show is the mitochondrial elements that are involved in cell death**. Initial production of mitochondrial ROS can lead to changes in membrane potential and opening of mitochondrial permeability transition pore (MPTP) opening. MPTP opening could further enhance mitochondrial ROS production, forming a vicious cycle that eventually leads to cell death and lung dysfunction.

### NF-κB pathway

The nuclear factor (NF)-*κ*B signaling pathway is involved in regulating gene expression in early inflammatory responses. NF-κB pathway is a primary target for activation by hyperglycemia, oxidative stress, and inflammatory cytokines [88]. The expression of TNF-α, IL-1β, IL-6 and other pro-inflammatory cytokines are regulated downstream of NF-*κ*B [89]. NF- κB has been shown to activate the genes encoding pro-inflammatory cytokines, cell adhesion molecules, nitric oxide synthase, and cyclooxygenase-2 [90]. In the lung tissues of diabetic rats, it has been reported that while the levels of IκB were declined when compared with that in the control group, the levels of phosphorylated I*κ*B and nuclear NF-*κ*B were actually elevated. Meanwhile, the mRNA levels and protein levels of iNOS and cyclooxygenase-2 were also up-regulated in the lung tissue of diabetic animals [91]. Additionally, protein carbonyl content was higher in diabetic lungs, but SOD and GSH activities were lower [22]. Hence, it seems that lungs are exposed to oxidative stress mediated by NF- κB activation in diabetes.

### Protein Kinase C pathways

Protein kinase C (PKC) is a family of enzymes that are involved in regulating the function of numerous proteins. PKC has been associated with vascular alterations such as increase in permeability, contractility, extracellular matrix synthesis, cell growth and apoptosis, angiogenesis, leukocyte adhesion, and cytokine activation and inhibition. It has been demonstrated that diabetic rats exhibited a significant decrease in LPS-induced phosphorylation of extracellular signal-regulated kinase, protein kinase C α and δ, p38, and protein kinase B [92]. Additionally, expression of iNOS and levels of IL-6 and cyclooxygenase are also decreased in the lung homogenates derived from diabetic rats [92]. In particular, PKC activation by hyperglycemia can activate NADPH oxidase that generates more ROS and accentuates oxidative stress [93]. Therefore, PKC dysregulation could be involved in diabetic lung injury.

## Role of mitochondrial dysfunction in diabetic lung injury

Mitochondria are important organelles for cell survival [94, 95]. Mitochondria play important roles in intracellular energy generation, in modulation of apoptosis, and in redox-dependent intracellular signaling [96]. Mitochondria can be both a source of ROS and a target of oxidative damage during oxidative stress. Hence, numerous studies attempting to link mitochondria to the complications of diabetes has focused on the role of mitochondrial ROS [97-100]. Prolonged hyperglycemia results in an over-generation of ROS by the mitochondria, which in turn may contribute significantly to the development of diabetic complications [100]. Interestingly, AMP kinase (AMPK) seems to be involved in the pathogenesis of diabetic complications. It has been reported in diabetes that C-peptide induces AMPKα activation and inhibits hyperglycemia-induced ROS production, mitochondrial membrane potential collapse, mitochondrial fission, and endothelial cell apoptosis [101]. It has also been demonstrated that increased mitochondrial ROS in diabetes is maintained by a feed-forward AMPK activation cascade [102]. Interestingly, basal ROS concentration was increased in lymphocytes from type 2 DM and triiodothyronine (T_3_) significantly stimulated ROS concentration in type 2 DM patients. Thus an increased mitochondrial sensitivity for T3 may be a significant factor responsible for increased ROS production in diabetic patients [103].

Moreover, hyperglycemia-induced over-production of mitochondrial ROS may lead to collapse of mitochondrial transmembrane potential and opening of the mitochondrial permeability transition pore (MPTP) [100, 104, 105]. The occurrence of apoptosis via cytochrome c release induced by MPTP opening may lead to more ROS generation [106, 107], forming a vicious cycle that eventually results in cell death [108] (Fig. 2). Conversely, therapeutic inhibition of mitochondrial ROS by antioxidants such as mito-TEMPO can decrease adverse cellular changes and mitigate cellular dysfunction in diabetic mice [109]. Thus, antioxidants targeting mitochondria could be an effective therapeutic approach for diabetic complications in the lung [110].

Based on research in other diabetic organs or tissues, we envision that defective or insufficient mitochondrial function plays potentially a pathogenic role in diabetic lung injury. Therefore, qualitative and quantitative analysis on functional perturbations of mitochondria needs to be conducted in the diabetic lung. In particular, the state of both mitochondrial transmembrane potential and the opening of MPTP in the diabetic lung injury need to be assessed.

## Summary

While numerous studies have shown that the lung is one of the target organs of diabetes, the biochemical pathogenesis of diabetic lung injury remains largely unexplored. In this review, we have summarized experimental and clinical evidence demonstrating diabetic lung dysfunction manifested by morphological and pathological changes. As sustained hyperglycemia could result in oxidative stress which contributes to tissue damage, oxidative stress has been postulated to be a major contributing factor in diabetic lung injury. Nonetheless, the role of mitochondria, a major source of ROS and oxidative stress, in diabetic lung injury has yet to be fully explored. Therefore, more studies should focus on mitochondrial dysfunction in the diabetic lung in the future, which will offer invaluable insights into design of therapeutic approaches for diabetic lung injury.
